# Botulinum Toxin in the Field of Dermatology: Novel Indications

**DOI:** 10.3390/toxins9120403

**Published:** 2017-12-16

**Authors:** Yoon Seob Kim, Eun Sun Hong, Hei Sung Kim

**Affiliations:** Department of Dermatology, Incheon St. Mary’s Hospital, College of Medicine, The Catholic University of Korea, Seoul 06591, Korea; kysbbubbu@hotmail.com (Y.S.K.); dmsun99@gmail.com (E.S.H.)

**Keywords:** botulinum toxin, biological effect, various cell types, neurotransmitter, dermatology, novel indication

## Abstract

Since its approval by the US Food and Drug Administration in 2002 for glabellar wrinkles, botulinum toxin (BTX) has been widely used to correct facial wrinkles. As a result, many consider BTX synonymous with cosmetic dermatology. Recent studies indicate that BTX elicits biological effects on various skin cell types via the modulation of neurotransmitter release, and it seems that BTX has a wider zone of dermatologic influence than originally understood. Clinicians and researchers are now beginning to explore the potential of BTX beyond the amelioration of facial lines and encouraging results are seen with BTX in a variety of skin conditions. In this paper, we review novel dermatological indications of BTX which includes (but not limited to) scar prevention, facial flushing, post-herpetic neuralgia and itch. These areas show great promise, but there is definite need for larger, double-blinded, randomized control trials against established treatments before BTX becomes a clinical reality.

## 1. Introduction

Botulinum toxin (BTX) is a potent neurotoxin produced by the bacterium *Clostridium botulinum.* Seven distinct isoforms (BTX-A, B, C, D, E, F, and G) have been described, with BTX-A and BTX-B being commercially available. BTX blocks the release of acetylcholine and a number of other neurotransmitters from presynaptic vesicles by deactivating SNARE proteins and has a long history of therapeutic application in neurological conditions with a strong efficacy and safety profile. As widely known, the skin interacts with the nervous system and there is increasing evidence that the neurological system directly participates in cutaneous inflammation and wound healing [1,2]. With that said, BTX has been used experimentally in a number of dermatological conditions which include scar prevention, facial flushing, post-herpetic neuralgia and itch with good results. The general mechanism which underlies these novel indications includes suppression of mast cell activity, and the inhibition of substance P, calcitonin gene-related peptide (CGRP) and glutamate release. In this review, we analyze the possible off-label applications of BTX based on published data.

## 2. Off-Label Use of BTX in Dermatology

### 2.1. BTX in Hypertrophic Scar Treatment

Scars are defined as marks that remain after the healing of a wound. They cause significant cosmetic concern, especially when located on conspicuous areas such as the head and neck. Hypertrophic scars and keloids represent an aberrant response to the wound healing process and are characterized by dysregulated growth and excessive collagen formation [3].

BTX has been reported as a treatment measure for hypertrophic scars and keloids in a number of studies [4,5,6,7] (Table 1). In one study [4], BTX injection (2.5 IU/cm^3^) was performed once a month for three months, leading to a significant decrease in erythema, itching, and pliability of the scar. In another study [7], 12 keloid patients received BTX injection (70–140 IU per session, every 3 months for a maximum of 9 months) and achieved more than 50% improvement in symptoms, size, height, and induration of the scar. In a randomized controlled trial (RCT) [5], the efficacy of BTX (5 IU/cm^3^, 3 sessions, repeated every 8 weeks) was compared with that of steroid injection (triamcinolone, kenacort 10 mg/cc, 6 sessions, repeated every 4 weeks) in keloids, where BTX led to a more significant reduction of subjective complaints (itch and pain of the scar).

The molecular mechanism of BTX on hypertrophic scars and keloids is not yet perfectly explained, but BTX has been shown to inhibit the proliferation of fibroblasts derived from hypertrophic scar tissues. In addition, BTX is reported to suppress the expression of transforming growth factor (TGF)-β1, collagen I and III, α-smooth muscle actin and myosin II protein in keloid fibroblasts [8,9,10,11].

One particularly favorable aspect of BTX is its ability to control the subjective symptoms of hypertrophic scars. BTX can immobilize the local muscles of a scar and reduce skin tension caused by the muscle pull [12]. This relieves trapped nerve fibers in keloids, neutralizing the itch and pain associated with small-fiber neuropathy [13]. Another advantage of BTX is the absence of skin atrophy and telangiectasia which is often seen after steroid injection.

The limitations of BTX on hypertrophic scars and keloids would be the high cost of the drug (with the dosages mentioned in prior studies) and its potential effect on the surrounding muscles. Due to these limitations, many suggest the use of BTX as an adjuvant rather than first line treatment for hypertrophic scars.

### 2.2. BTX in Scar Prevention

Nowadays, many acknowledge the role of active scar prevention important in post-operative scar management. A key factor that determines the final cosmetic appearance of a surgical scar is the tension that acts on the wound edges during the healing phase [14,15]. By blocking acetylcholine neurotransmitter release from peripheral nerves, BTX allows near-complete elimination of dynamic muscle tension on the healing wound. The tension relieving properties, together with the direct inhibitory effects of BTX on fibroblasts and TGF-β1 expression support its usage in surgical scar prevention [16,17,18]. The anti-inflammatory effect of BTX and its action of the cutaneous vasculature calms down the inflammatory phase (immediate to 2–5 days) of the wound healing process which may also contribute to scar prevention.

A number of studies have reported the effectiveness of BTX in scar prevention [19,20,21,22] (Table 2). In a split-scar RCT [19], the safety and efficacy of early postoperative BTX injection was assessed in 15 thyroidectomy scar patients. A single treatment with either BTX (20–65 IU) or 0.9% saline (control) was applied to fresh scars (within 10 days of thyroidectomy), where the BTX-treated halves showed a significantly better outcome in terms of scar scales and patient satisfaction compared to the saline treated sides. In 2006, Gassner [21] tested whether postoperative injection of BTX improved facial scars following forehead lacerations and excisions. BTX (15–45 IU) was injected to post-op scars within 24 h after wound closure to produce enhanced wound healing and improved cosmesis compared to placebo (normal saline) injection.

BTX is best used for op scars. It would be optimal to inject BTX intraoperatively or shortly (preferably within days) after the surgery. To note, BTX should be avoided in open wounds as it delays wound closure.

### 2.3. BTX in Rosacea and Facial Flushing

Rosacea is a common inflammatory dermatosis characterized by persistent erythema, telangiectasia, papules, pustules, and facial flush. Oral medication, topicals, and laser therapy are routinely performed but often fail to relieve the facial flush. Persistent facial flushing is also a troublesome menopausal symptom.

A number of reports demonstrate the possible action of BTX on rosacea and menopausal hot flashes [23,24,25,26] (Table 3). In a prospective pilot study [23], the effect of BTX on the Dermatology Life Quality Index (DLQI) of patients with facial flushing was examined. BTX was injected once up to a total dose of 30 units on the cheeks which led to a significant decrease in DLQI at 2 months follow-up. Odo et al. [26] reported BTX (6.2 IU of abo-BTX per injection point, 40 points over the face, chest, neck, and scalp) to significantly reduce the mean number of menopausal hot flashes at day 60. The effect of abo-BTX was also investigated in 15 patients with rosacea. 15–45 IU of BTX was injected to the face which resulted in a statistically significant improvement in erythema at 3 months follow-up [24]. Adverse effects were rarely reported in the studies.

One possible mechanism by which BTX improves flushing is the potent blockade of acetylcholine release from autonomic peripheral nerves of the cutaneous vasodilatory system [27,28]. It is also well-known that BTX inhibits the release of inflammatory mediators such as substance P and calcitonin gene-related peptide (CGRP) [29]. The reduction and control of local skin inflammation may allow the erythema to fade out.

Larger, controlled, randomized studies are warranted to determine the optimal dose and duration of BTX activity on rosacea and facial flushing. BTX injection for facial flushing has additional benefits as it also improve the fine lines and wrinkles by diminishing the pull of the facial depressors.

### 2.4. BTX in Postherpetic Neuralgia

Postherpetic neuralgia (PHN) is the most frequent chronic complication of herpes zoster and the most common neuropathic pain resulting from infection. It is conventionally defined as dermatomal pain (usually a score of 40 or higher on a Likert scale ranging from 0-no pain to 100-worst possible pain), persisting at least 90 days after the appearance of the acute herpes zoster rash. PHN causes considerable suffering and results in a health care burden at both the individual and societal levels [30].

Treatment approaches include nonsteroidal anti-inflammatory drugs, gabapentin, opioids, and tricyclic antidepressants as well as topical anesthetics and capsaicin cream, but pain can be resistant to all of these drugs.

A number of reports have been made on the efficacy of BTX in PHN [31,32,33] (Table 4). Xiao et al. [33] performed a randomized, double-blind, placebo-controlled study on 60 PHN patients with the following arms: the BTX group, the 0.5% lidocaine group, and the 0.9% saline group. All patients were treated once (as for BTX, a dose of 200 IU at maximum), and were followed-up for 3 months. The BTX treated patients were found to have the most significant improvement in Visual Analog Scale (VAS) and sleep quality compared to those of the other two groups. Apalla et al. [32] also performed a RCT where 30 PHN patients received either BTX (200 IU in total) or placebo. BTX patients showed a significant reduction in VAS pain scores as well as the sleep scores which lasted for approximately 16 weeks. In a prospective study, Ding et al. [31] treated 58 PHN with BTX (50 to 100 IU in total) to find promising results (reduced frequency of pain attacks, lower pain severity, reduction in the quantity of painkillers consumed by patients) with very few adverse reactions.

The mechanism involved in the pain-relieving effect of BTX is still unclear, but it is thought that both the peripheral and central mechanism play a role [33]. The peripheral effects of BTX injection come through the inhibition of neuropeptide release from the peripheral nociceptive nerves [34,35]. In addition, BTX has been suggested to exert central nervous system (CNS) effects through axonal transport to the CNS after peripheral application [36,37].

Although promising, cost would be one of the main considerations to BTX use in PHN. Also, unlike other therapeutic modalities, BTX induces antitoxin antibodies which can limit the clinical effectiveness of the drug after repetitive, long-term use.

### 2.5. BTX in Pruritus

Pruritus (also known as itch) is an unpleasant sensation of the skin leading to the desire to scratch. Among the 4 subtypes (pruriceptive, neurogenic, neuropathic, and psychogenic itch), the pruriceptive itch is a peripherally induced pruritus arising from the skin and mucosa and is often seen in dermatological disease.

A number of reports have been made on the efficacy of BTX in pruriceptive pruritus [38,39,40] (Table 5). Recalcitrant pruritus is a hallmark of lichen simplex, a localized variant of atopic dermatitis. In an open pilot study [40], BTX (abo-BTX, 20–80 IU) was injected intradermally into 5 circumscribed lichenoid lesions with recalcitrant pruritus. Within a week, all patients reported to have noticeable alleviation of itching and at 12 weeks, all were still free from the uncontrollable urge to scratch. Itch is also a common and well-recognized problem in burns [38]. Nine patients with recalcitrant itching secondary to burns were treated with BTX (dosage not specified) where the burn itch fell to 0 out of 10 in 4 weeks. The average duration of symptom free period was reported as nine months.

As clinical evidence has revealed the antipruritic effect of BTX, Arendt-Nielsen et al. [39], investigated the effect of subcutaneous administration of BTX on experimentally histamine-induced itch in human skin. In this double-blind, placebo-controlled study, 14 healthy men received BTX and isotonic saline on the volar surface of either forearm. Histamine prick tests were performed four times at the treatment sites (before treatment, and days 1, 3, and 7 after treatment) where BTX reduced the histamine-induced itch intensity, and itch area compared with saline at all time points.

Several possible mechanisms can be responsible for the reduction of pruriceptive itch. Acetylcholine mediates itch in pruritic skin conditions such as atopic dermatitis [41] and BTX inhibits the release of acetylcholine from presynaptic vesicles [42]. BTX is also known to interact with molecules associated with itch and flare such as substance P (releases histamine via the activation of mast cells, promotes vasodilation) and CGRP (a potent vasodilator) [36,43,44,45]. BTX inhibits the release of such mediators, thus reducing the sensation of itch. Lastly, BTX has also been shown to stabilize mast cells and inhibit their degranulation [46].

Pruritogenic pruritus is usually accompanied by skin inflammation. Since BTX is capable of reducing neurogenic inflammation [29], it is natural to expect improvement of the primary skin disease (e.g., atopic dermatitis, psoriasis) as well, which has in fact been reported through animal studies [47,48], human studies [49] and case reports [50,51]. Although promising in pruritogenic itch (and also in inflammatory dermatoses), we feel that BTX would be best used as an adjunct to conventional therapy. It should be applied focally, considering that the product can induce muscle weakening.

### 2.6. BTX in Dermatological Conditions Associated with Hyperhidrosis

A number of skin disease are caused by and/or have symptoms that are exacerbated by hyperhidrosis, a condition that can be treated successfully with BTX.

Pompholyx or dyshidrotic eczema is a common vesiculo-bullous disease of the palms and/or soles. A hallmark of this disease is its tendency to relapse in response to various provoking factors which includes wet work, occlusion and hyperhidrosis. An intra-individual study of 10 patients [52] (Table 6) investigated the use of BTX (mean dose of 162 IU per palm) for pompholyx, using the using the untreated site as control. 70% of patients reported a marked improvement of both sweating and itching on the treated site after 6 weeks. In another side-by-side trial [53], dyshidrotic hand eczema was treated with BTX (100 IU per palm) as an adjunct to topical steroids. Six patients who completed the study were found to have improved symptoms of pompholyx and reduced number of relapses by BTX injection.

The anhidrotic effect of BTX in pompholyx can be explained by its action on smooth muscles surrounding the sweat glands and through the inhibition of acetylcholine release. Inhibition of substance P release also explains the reduction in pruritus [54,55].

Hidradenitis suppurativa (HS) is a chronic inflammatory dermatosis of the apocrine glands which typically affects the axillae and groin. Patients afflicted by HS have severe discomfort and treatment is extremely challenging. It is well-known that a moist environment in folds, especially in the axilla and groin, provides ideal conditions for the flourishing of bacteria and is a precipitating factor of HS.

In 2005, HS on the axillae was first reported to be successfully treated with BTX (abo-BTX, 250 IU) with 10 months of complete remission [56] (Table 6). Khoo et al. [57] also confirmed the efficacy of BTX in HS where a 46-year-old woman with Hurley stage 2 HS responded well to axillary BTX treatment (50 IU per side) with a remission period of 12 months. The patient had been recalcitrant to conventional treatments and also underwent surgical drainage.

The exact mechanism by which BTX affects the disease process in HS is unclear but it is likely that the effect of BTX on sweat production reduces the population of skin flora and its potential inflammatory effect [56,57]. A second hypothesis is that by inhibiting apocrine secretion, BTX prevents the rupture and spread of follicular material from the pilosebaceous unit [57].

BTX has been studied in inverse psoriasis (Table 6) which is also thought to be exacerbated by excessive sweating. A pilot study of 15 patients with flexural psoriasis [49] showed that 50–100 IU of BTX improved subjective symptoms and objective photographic evidence of disease in 87% of patients at 2, 4, and 12 weeks follow-up. It is hypothesized that the beneficial effects of BTX in inverse psoriasis is largely due to the reduction of local sweating in folds [49]. Patients with psoriasis are also known to have a higher concentration of substance P receptors in their skin [58,59], meaning that BTX can reduce pruritus and vasodilation by inhibiting neuropeptide liberation (and preventing substance P binding to multiple receptors).

Hailey–Hailey disease is an autosomal dominant acantholytic disorder with mutation of the *ATP2C1* gene, clinically manifesting as macerated flexural erythema. Heat and sweat aggravate the disease, worsening the discomfort and pruritic symptoms.

Several case reports [60,61] (Table 6) have evidenced improvement of Hailey–Hailey disease with the use of BTX (50–125 IU per side). In one study, the effect of BTX was found to be comparable to that of laser ablation and dermabrasion [61].

BTX can rationally ameliorate the symptoms of Hailey–Hailey disease via its inhibition of acetylcholine and substance P release from the nerve endings [54,55] (Table 6). Although more clinical evidence is needed to prove effectiveness, BTX may be considered as a possible treatment modality for Hailey–Hailey disease recalcitrant to conventional treatment.

### 2.7. BTX in Oily Skin

Sebum contributes to the delivery of fat-soluble antioxidants to the skin surface and has antimicrobial activity, thereby functioning as a skin barrier. However, excess sebum blocks the pores, provides nourishment to bacteria, and can result in skin inflammation (e.g., acne, seborrheic dermatitis).

Recently, insights into the effect of BTX on sebum production have been published [62,63] (Table 7). Min et al. [62] randomly assigned 42 volunteers with forehead wrinkles to receive 10 or 20 units of BTX, which was administered in five standard injection sites. Treatment with BTX exhibited significant sebum reduction at the injection site of both groups, with a sebum gradient surrounding the injection point. The efficacy did not improve significantly with higher injection doses and the sebum production recovered to normal levels at 16-week follow-up for both treatment groups. Rose and Goldberg [63] also evaluated the safety and efficacy of BTX on the oily skin of 25 subjects. A 10-point injection was made with BTX (abo-BTX, total amount of 30-45 IU) on the forehead to find significantly lower sebum production and high patient satisfaction.

The mechanism by which intradermal BTX injection results in decreased sebum production is not entirely clear because the role of the nervous system and acetylcholine on sebaceous glands is not well defined. However, it is most likely that the arrector pili muscles and the local muscarinic receptors in the sebaceous glands are targets for the neuro-modulatory effects of BTX. Li et al. [64] demonstrated that nicotinic acetylcholine receptor α7 (nAchRα7) is expressed in human sebaceous glands in vivo, and acetylcholine signal increased lipid synthesis in vitro in a dose-dependent manner. Further study is needed to determine the best candidates, optimal injection techniques and doses.

## 3. Conclusions

In this review, we highlighted the promising outcomes of BTX in several off-label indications of interest for dermatologists. There is overwhelming evidence that BTX exhibits biological effects on many human cell types, but much is yet to be learned about the drug and its mechanism of action. Knowing that the skin closely interacts with the nervous system, future studies should investigate the link between BTX and the cutaneous neuroimmune system to better understand its therapeutic potential in dermatology. A consensus on the dose regimen and injection technique is also desirable for standardized treatment. Generally, high doses of BTX were applied, with an average total of 300 IU for hypertrophic scars, 50 IU for scar prevention, 50–100 IU for facial flush/rosacea, 100 IU for PHN, 150 IU for pompholyx, 100 IU for HS, 75 IU for inverse psoriasis and 250 IU for Hailey-Hailey disease. Lastly, with the limitations of BTX treatment (high cost, muscle weakening, risk of tachyphylaxis and production of antibodies), BTX may be optimally used as an adjunct in recalcitrant cases to conventional therapy.

## Figures and Tables

**Table 1:** Representative studies of botulinum toxin (BTX) in hypertrophic scar treatment.

First Author [Ref.], Year	Type of Study	*n*	Treatment Regimen	Outcome Measures	Follow-Up	Results (Including Adverse Effects)
Elhefnawy [4], 2016	Prospective, single arm (BTX)	20	BTX once a month for 3 months. BTX concentration: 5 IU/0.1 mL; Injected dose: 2.5 IU/cm^3^, not exceeding 100 IU/session	Overall assessment made by the patient and physician (5-point scale). Lesions were assessed for erythema, itching, and pliability; each item was assessed on a 5-point scale	6 months	Therapeutic satisfaction was “good” in 14 patients, “excellent” in 6; The mean erythema score decreased from 3.2 to 1.0, mean pliability score from 3.3 to 0.8 and the mean itching score from 2.7 to 0.7; All findings were statistically significant. No recurrence or complications.
Shaarawy [5], 2015	Randomized, double-blinded, comparative study (BTX vs. IL steroid injection)	24	Group A (12 patients; Triamcinolone, Kenacort^®^ 10 mg/cc; repeated every 4 weeks for 6 sessions/or till complete remission) Group B (12 patients; BTX 5 IU/cm^3^ repeated every 8 weeks for three sessions/or till complete remission of keloid)	Objective parameters (hardness, elevation, and redness) and subjective complaints (itching, pain and tenderness) on a scale of 0–3; Volume of keloid; Patient satisfaction (3-point scale)	7 months	Significant decrease in scar volume after treatment with a volume reduction of 82.7% (group A) and 79.2% (group B). Significant softening, significant decrease in height and significant decrease in redness with little difference between the groups. All patients mentioned a significant reduction of their subjective complaints which was more prominent in group B (BTX group). Skin atrophy and telangiectasia was evident in 3 patients of group A.
Xiao [6], 2009	Prospective, single arm (BTX)	19	BTX once a month for a total of 3 months. BTX injection dose: 2.5 IU/cm^3^, not exceeding 100 IU/session.	Physician and patient satisfaction (5-point scale); Clinical symptoms in terms of erythema, pliability and itching sensation (each graded on a 5-point scale)	6 months	Patient satisfaction: good (63.1%), excellent (36.8%) Physician satisfaction: good (78.9%), excellent (10.5%) Mean erythema score decreased from 3.41 to 1.23; The pliability score decreased from 3.85 to 0.78; and the itching score decreased from 3.50 to 0.83. All reductions were statistically significant. Besides the injection pain, no other complication was detected in this study.
Zhibo [7], 2009	Prospective, single arm (BTX)	12	BTX at 3 months interval for a maximum of 9 months. BTX concentration: 35 IU/mL; injected dose: 70–140 IU/session	Improvement was judged based on a decrease in size and flattening of the lesion with a 5-point scale; Patient satisfaction	1 year	Therapeutic outcome: excellent (25%), good (41.7%), fair (33.3%). The level of patient satisfaction was very high. There were no serious adverse sequelae.

BTX: Onabotulinum toxin unless otherwise stated, IL: intra-lesional.

**Table 2:** Representative studies of BTX in scar prevention.

First Author [Ref.], Year	Type of Study	*n*	Treatment Regimen	Outcome Measures	Follow-Up	Results (Including Adverse Effects)
Kim [19], 2014	A split-scar, double-blind, RCT (BTX vs. saline)	15	Treatment with either BTX or 0.9% normal saline on scar halves. A single treatment delivered within 10 days of thyroidectomy. BTX concentration: 5 IU/mL; Injection dose: 20–65 IU	Modified Stony Brook Scar Evaluation Scale (SBSES) Patient satisfaction (4-point scale)	6 months	A significant improvement in SBSES score was noted for the BTX-treated halves (*p* < 0.001), with minimal change on the saline-treated side. The mean calculated difference in SBSES scores (final/initial) between the BTX-treated side and the saline-treated side was also significant (*p* < 0.001). Subjects were significantly more satisfied with the overall outcome of the BTX-treated side at 6 months’ follow-up, according to a four-point grading scale (*p =* 0.000; 95% CI 1.24 to 2.36)
Ziade [20], 2013	RCT (BTX vs. no injection)	BTX group: 15 (4 lost for FU) Control group: 15 (2 lost for FU)	BTX group: A single treatment delivered within 72 h following op. BTX concentration: 10 IU/mL; Injection dose: 15–40 IU. Control group: No injection	Patient Scar Assessment Scale (PSAS) Observer Scar Assessment Scale (OSAS) Vancouver Scar Scale (VSS), Visual Analogue Scale (VAS)	12 months	No statistically significant differences were found between the two groups for the PSAS, OSAS and VSS scores. The median VAS rated by the six evaluators was 8.25 for the botulinum toxin-treated group compared with 6.35 for the control group (*p* < 0.001).
Gassner [21], 2006	RCT (BTX vs. saline)	BTX group: 22 (6 excluded) Control group: 20 (5 excluded)	BTX group: A single injection within 24 h after wound closure. BTX concentration: 75 IU/mL; Injection dose: 15–45 IU. Control group: A single injection within 24 h after wound closure. Injection dose: 0.2–0.6 mL of saline	Visual Analogue Scale (VAS)	6 months	The overall median VAS score for the BTX-treated group was 8.9 compared with 7.2 for the placebo group (*p* = 0.003), indicating enhanced healing and improved cosmesis of the experimentally immobilized scars.
Wilson [22], 2006	Prospective, single-arm	55 (15 dropped out)	BTX was injected once at the end of the operation. BTX concentration: 10 IU/mL; Injection dose: 1.5 IU per cm of wound length.	Objective assessment Subjective assessment	12–16 months	The outcome was considered highly satisfactory in 36 patients (90%). Thirty patients rated the improvement as marked (75%), six rated it as significant (15%), and four rated it as unchanged (10%). In 3 cases (7.5%), the scars re-widened, while in one case (2.5%), residual scar depression persisted.

BTX: Onabotulinum toxin unless otherwise stated.

**Table 3:** Representative studies of BTX in rosacea and facial flushing.

First Author [Ref.], Year	Type of Study	*n*	Treatment Regimen	Outcome Measures	Follow-Up	Results (Including Adverse Effects)
Eshghi [23], 2016	Prospective single arm (BTX)	24	A single treatment with BTX. 1 IU of BTX was injected intracutaneously in every square cm, to a total dose of 30 IU on both sides of cheeks.	The Dermatology Life Quality Index (DLQI)	2 months	The mean of DLQI improved from 8.08 ± 1.17 (baseline) to 4.5 ± 1.21 (2 months follow-up) (*p* < 0.005)
Bloom [24], 2015	Prospective, single arm (Abo-BTX)	25 (15 completed the study)	A single treatment with abo-BTX. Abo-BTX concentration: 100 IU/mL; Injected dose: 15–45 IU to the nose, cheeks, forehead, and chin	Facial erythema assessed by a non-treating physician using a standardized grading system (0–3)	3 months	The treatment resulted in statistically significant improvement in erythema grade at 1 (*p* < 0.05), 2 (*p* < 0.001), and 3 months (*p* < 0.05) after treatment when compared with baseline.
Geddoa [25], 2013	Prospective, single arm (BTX)	22 (18 included in the final analysis)	A single treatment with BTX for 20 patients and two sessions of treatment for 2. BTX concentration: 10 IU/mL; Injected dose: 1–2 IU/cm^2^ with maximum total dose of 100 IU (neck and/or chest)	Dermatology Life Quality Index (DLQI)	4 weeks	The mean change in DLQI (before-after treatment) was 3.56 ± 4.6, suggesting a significant improvement in quality of life at 4 weeks following treatment (*p* < 0.004)
Odo [26], 2011	RCT (Abo-BTX vs. saline)	60 women with menopausal hot flushes Group BTX: 30 (25 completed) Group Control: 30 (23 completed)	A single treatment with either abo-BTX or saline. Group BTX: Abo-BTX concentration: 500 IU/3.2 mL; Injected dose: 6.2 IU at each selected point in the skin (40 injection points to the face, chest, neck, and scalp) Group Control: saline solution injected at a volume of 0.04 mL per injection point	Intensity of sweating, number of hot flashes, and Starch-Iodine test. Number of women reporting episodes of night sweating and mood changes	6 months	The sweating and hot flashes were less severe than before abo-BTX treatment, especially at 2 months follow-up; In the control group, there was no significant difference in mean intensity of sweating or in the mean number of hot flashes. Other menopausal symptoms such as night sweats were found better 2 months after abo-BTX treatment than in the control group (*p* < 0.001).

BTX: Onabotulinum toxin unless otherwise stated, Abo-BTX: Abobotulinum toxin.

**Table 4:** Representative studies of BTX in postherpetic neuralgia (PHN).

First Author [Ref.], Year	Type of Study	*n*	Treatment Regimen	Outcome Measures	Follow-Up	Results (Including Adverse Effects)
Ding [31], 2017	Prospective, single arm (BTX)	58	A single session of treatment was performed. BTX concentration: 4 IU/mL; Injection dose: 50–100 IU in total	Pain severity (VAS) Neuropathy pain scale (NPS) Quality of Life Scale (SF-36) PHN seizure severity, seizure duration, and frequency of attacks. The use of painkillers	6 months	At 6 months follow-up, a significant decrease in seizure frequency, seizure duration, VAS score, NPS score, SF-36 score and the required amount of painkiller was observed (*p* < 0.05). After BTX injection, 4 patients complained of pain around the injection area which disappeared within a week.
Apalla [32], 2013	Double-blinded RCT (BTX vs. saline)	BTX group: 15 Control group: 15	BTX group: A single treatment delivered. BTX concentration: 25 IU/mL; Injection dose: each patient received 40 injections in total (5 IU/point). Control group: A single treatment with saline.	Pain severity (VA) Quality of sleep	4 months	Thirteen patients from the experimental arm achieved at least 50% reduction in VAS score, compared with none of the placebo group (*p* < 0.001). BTX patients showed significant reduction in VAS pain scores between baseline and week 2, which persisted for a median period of 16 weeks. BTX patients showed significant reduction in sleep scores between baseline and week 2, which remained unchanged until week 16 (*p* < 0.001). Treatment was well-tolerated.
Xiao [33], 2010	Double-blinded RCT (BTX vs. 0.5% lidocaine vs. saline)	BTX group: 20 (19 completed) Lidocaine group: 20 (19 completed) Control group: 20 (18 completed)	BTX group: A single injection. BTX concentration: 5 IU/mL; Injection dose: 200 IU at maximum. Lidocaine group: A single session of treatment of the same volume as BTX. Control group: A single injection of the same volume as BTX	Visual Analogue Scale (VAS) Quality of Life Percent of Opioid use	3 months	Compared with pretreatment, VAS pain scores decreased at day 7 and 3 months posttreatment in all 3 groups. However, the VAS pain scores of the BTX group decreased more significantly compared with lidocaine and placebo groups at day 7 and 3 months posttreatment (*p* < 0.01). Sleep time improved in all 3 groups but was most significant in the BTX group compared with the lidocaine and placebo groups (*p* < 0.01). The percentage of subjects using opioids posttreatment in the BTX was the lowest (21.1%), compared with the lidocaine (52.6%) and placebo (66.7%) groups (*p* < 0.01).

BTX: Onabotulinum toxin unless otherwise stated, PHN: post-herpetic neuralgia.

**Table 5:** Representative studies of BTX in itch.

First Author [Ref.], Year	Type of Study	*n*	Treatment Regimen	Outcome Measures	Follow-Up	Results (Including Adverse Effects)
Akhtar [38], 2012	Prospective, single arm (BTX)	9	Treatment with BTX once on the burn scar. BTX concentration: 10–25 IU/mL; Injection dose: not stated	Severity of itch (0–10 scale)	11.3 months on average	On average, the burn covered 24% of the total body surface area and 87.5% of patients rated their burn itch as being severe (>7 on the itch scale). Following the administration of BTX, this fell to 0 out of 10 in 4 weeks. The average duration of symptom free period was 9 months (3–18 months).
Gazerani [39], 2009	Double-blind, split arm, RCT (BTX vs. saline)	14	BTX treated arm: 5 IU of BTX was injected into the skin once. Control arm: The same volume of 0.9% saline as in the BTX treated side was injected. After BTX and saline injection, a skin prick test with histamine was performed on both sites.	Itch ratings (0–10 scale), itch area, neurogenic inflammation (visible flare area) Blood flow (Laser Doppler imaging) Cutaneous temperature (Infrared thermography	1 week	BTX reduced the histamine-induced itch intensity (*p* < 0.001), and itch area (*p* = 0.011) compared with saline at all time points after treatment. The duration of itch was also shorter for BTX treated areas (*p* < 0.001), with a peak effect at day 7. The flare area was smaller in the BTX treated arm compared with the saline treated arm at all time points after treatment (*p* = 0.002). Findings from blood flow (*p* < 0.001), and temperature measurements (*p* < 0.001), clearly showed suppressive effects of BTX on vasomotor reactions, with the maximal effect on day 3 and 7.
Heckmann [40], 2002	Prospective, single arm (Abo-BTX)	4	Abo-BTX was injected to a total of 6 lichen simplex chronicus (LSC) patches once. Abo-BTX concentration: 100 IU/mL; Injection dose: 20–80 IU.	Sensation of pruritus (VAS 0–10)	4 months	After a week, all patients reported a noticeable alleviation of itching. Three patients felt no more itching at all; in one patient pruritus was reduced to less than 50% according to the VAS used before and after treatment. After 4 weeks, 5 of 6 lesions had cleared without any other treatment. After 12 weeks, 3 patients were still free of symptoms. One patient who had one lesion on the shin developed a new lesion on the dorsum of his foot which was cleared in 2 weeks after BTX treatment.

BTX: Onabotulinum toxin unless otherwise stated, Abo-BTX: abobotulinumtoxin, VAS: Visual Analogue Scale.

**Table 6:** BTX in dermatologic disease associated with hyperhidrosis.

First Author [Ref.], Year	Type of Study	*n*	Treatment Regimen	Outcome Measures	Follow-Up	Results (Including Adverse Effects)
**Pompholyx**						
Swartling [52], 2002	A side-by-side prospective controlled trial (BTX vs. no treatment)	10	BTX group: BTX was injected once. BTX concentration: 100 IU/mL; Injection dose: a mean of 162 IU. Control group: No treatment.	Effect of treatment (5-point scale) VAS for itch Disease activity score Extent of the dermatitis	5–6 weeks	In the self-assessment, 7 of the 10 patients in the study experienced good or very good effect of the treatment. After injection with BTX, the VAS score for itching decreased by 39% on the treated side compared to an increase by 52% on the untreated side. Comparing treated vs. untreated sides, it was found that with BTX injection, a decrease in the disease activity score (54% vs. 29%), occurrence of vesicles (74 vs. 27), infiltration (54 vs. 18), erythema (53 vs. 30), and extent of the disease (58 vs. 31). No changes or only minor changes were seen in the objective parameters of scaling, crusting, and excoriations.
Wollina [53], 2002	A side-by-side prospective controlled trial (BTX vs. control)	8 (6 completed the study)	Topical steroid was applied to both hands in combination with BTX on one hand and no additional treatment on the other. A single injection of BTX was given. BTX concentration: 50 IU/mL; BTX was injection in aliquots of 5 IU per point; Injection dose: Not mentioned.	Dyshidrotic Eczema Area and Severity Index (DASI)	8 weeks	Six patients completed the study. The mean DASI score changed from 28 to 17 with topical therapy alone and from 36 to 3 with adjuvant BTX (*p* < 0.01). Itching and vesicles were inhibited earlier when using the combination of steroids and BTX. There was one relapse in the steroid group and none in the BTX group.
**Hidradenitis suppurativa**						
Khoo [57], 2014	Case report (BTX)	3 (only one described in detail)	Over the course of 3 years, a Hurley Stage II HS patient received 4 BTX treatments. 50 IU of BTX administered to each axilla per treatment. BTX concentration: 25 IU/mL			The patient showed good clinical response within 3 months of her first treatment, and, following her second treatment, went into clinical remission. She was still in remission when discharged from follow-up 1 year after her fourth treatment.
O’Reilly [56], 2005	Case report (Abo-BTX)	1	BTX was injected once to both axilla. Injection dose: 250 IU of abo-BTX in total.			There was no evidence of active inflammation on follow-up at a fortnight after administration. The patient had complete remission of symptoms until approximately 10 months later, when the first symptoms of mild inflammation re-appeared.
**Psoriasis**						
Zanchi [49], 2008	Prospective, single-arm (BTX)	15	BTX was injected once to the inverse psoriasis sites. BTX concentration: 20 IU/mL; Dose injected: 50–100 IU in total.	Photographic assessment of the psoriatic area Subjective symptomatology (10-point VAS)	12 weeks	The location of the psoriasis was as follows: armpits (7 patients), sub-mammary sulcus (6), intergluteal folds (7), inguinal folds (5) and umbilicus (1). Subjective symptomatology according to the 10-point VAS scale improved in all patients. Mean VAS scores were 9.1 at the pre-treatment assessment, with post-treatment mean scores of 4.2 after 2 weeks, 2.1 after 4 weeks and 2.4 after 12 weeks. Erythema extension, intensity and infiltration improved in 13 of 15 patients (87%). The change in the erythematous area was evident from the first post-treatment assessment at 2 weeks and continued to improve until the assessment at 4 weeks. At the final visit (12 weeks post-treatment), improvement had been maintained.
**Hailey–Hailey disease**						
Lopez-Ferrer [60], 2012	Case report (BTX)	3	Case 1: 80 IU of BTX was first administered on each axilla. A total of 200 IU of BTX was injected every 2 months for maintenance. Case 2: 80–300 IU of BTX was injected to the groin, below the left breast, the left axilla, and the side of the neck. Case 3: 200–300 IU of BTX was injected to the axilla, sub-mammary region, and groin.			The Hailey–Hailey disease improved in all 3 patients after BTX injection. BTX injection had to be repeated for maintaining remission.
Konrad [61], 2001	Case report with side-by side comparison (BTX alone vs. BTX + Erbium: YAG vs. BTX + dermabrasion	1	Both sub-mammary areas were treated with BTX at a concentration of 20 IU/mL. Four days later, surgical (right side) or laser therapy (left side) was performed on an area of 5 × 5 cm^2^.			Wound healing was faster after laser (7 days) versus dermabrasion (14 days). Those areas treated only with BTX showed remission of hyperhidrosis within 3 days and clearance of Hailey-Hailey within 2 weeks. During a follow-up of 12 months, no relapse was seen for dermabrasion, laser ablation and BTX. Final cosmetic results were comparable.

BTX: Onabotulinum toxin unless otherwise stated, Abo-BTX: Abobotulinumtoxin, VAS: Visual Analogue Scale.

**Table 7:** Representative studies of BTX in oily skin.

First Author [Ref.], Year	Type of Study	*n*	Treatment Regimen	Outcome Measures	Follow-Up	Results (Including Adverse Effects)
Min [62], 2015	Prospective (BTX 10 IU vs. BTX 20 IU)	42 (41 completed the study) 20 received 10 IU of BTX 20 received 20 IU	Treatment with BTX once on the forehead. BTX concentration: 40 IU/mL; Injection dose: A final volume of 10 IU or 20 IU was injected evenly in 5 injection sites.	Sebum production (sebumeter)	16 weeks	Treatment with BTX exhibited significant sebum alteration at the injection site of both groups (10 IU, 20 IU), with a sebum gradient surrounding the injection point. The efficacy did not improve at higher injection doses, with the four-unit regimen generally not being more potent than the two-unit regimen. The sebum production recovered to normal levels at the 16-week follow-up for both treatment groups, indicating that a higher dosage (4 units) did not results in a longer duration until relapse compared with the two-unit dose.
Rose [63], 2012	Prospective, single-arm (Abo-BTX)	25	Abo-BTX was injected once on the forehead. Abo-BTX concentration: 100 IU/mL; Injection dose: A total of 30–45 IU delivered to 10 injection sites.	Sebum production (sebumeter) Patient satisfaction (4-point scale)	3 months	Treatment with BTX resulted in significantly lower sebum production at 1 week and 1, 2, and 3 months after injection (*p* < 0.001). Twenty-one patients (91%) reported that they were satisfied (50–75% improvement) with intradermal BTX as a treatment for oily skin.

BTX: Onabotulinum toxin unless otherwise stated, Abo-BTX: abobotulinumtoxin.
